# Capsular Typing and Molecular Characterization of *Streptococcus agalactiae* Strains Isolated From Bovine Mastitis in Iran

**DOI:** 10.1002/vms3.70275

**Published:** 2025-03-20

**Authors:** Pegah HajiAhmadi, Hassan Momtaz, Elahe Tajbakhsh

**Affiliations:** ^1^ Department of Microbiology Shahrekord Branch Islamic Azad University Shahrekord Iran

**Keywords:** antibiotic resistance profile, bovine mastitis, capsular serotypes, streptococcus agalactiae

## Abstract

*Streptococcus agalactiae* infections may cause clinical or subclinical mastitis in dairy cows by invading the mammary gland. This research included the isolation of 29 strains of *S. agalactiae* from 425 milk samples obtained from cows affected by clinical mastitis in Chaharmahal and Bakhtiari province, Iran. The antimicrobial sensitivity of *S. agalactiae* strains was determined using 16 antibiotics from seven different classes. The epidemiological spread of *S. agalactiae* was determined by identifying the serotypes of isolates using multiplex PCR. In addition, the presence of antibiotic‐resistance genes and virulence genes were investigated to infer the pathogenicity and antibiotic resistance of *S. agalactiae* using the multiplex PCR method. A total of 29 strains of *S. agalactiae*, which constitute 6.82% of the samples, were identified based on phenotypic traits, biochemical properties and *dltR* gene amplification. Multiplex serotype polymerase chain reaction study showed that most of the isolates belonged to Type III serotype. Phenotypically, 100% of the isolated strains were resistant to tetracycline and penicillin. The frequency of resistance to beta‐lactams (penicillin and amoxicillin) was 100% and 82.75%. *tetM*, *tetO* and *tetT* genes, responsible for resistance to tetracyclines, were found in all samples, corresponding to the drug‐resistant phenotype. Among the genes related to the virulence factor, 100% of the isolates had the *dlts* gene. The examination of virulence genes revealed that the majority of isolates included the *cfb*, *pavA* and *scPb* genes. This data has the potential to assist in the prevention and management of mastitis and enhance our comprehension of epidemiological patterns in dairy cows affected by *S. agalactiae* in Chaharmahal and Bakhtiari province.

## Graphical abstract

The following three main findings were obtained from the present study:

1‐ The most common capsular serotype identified in *Streptococcus agalactiae* strains isolated from bovine mastitis cases was serotype Number III.

2‐ Most of the *Streptococcus agalactiae* strains isolated from bovine mastitis were antibiotic resistant to antibiotics commonly used in the treatment of infectious diseases in cattle.

3‐ Most of the *Streptococcus agalactiae* strains isolated from bovine mastitis had virulence factors, especially *dlts* and *cfb* genes.

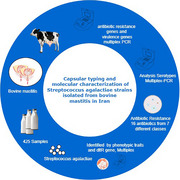

## 1 Introduction

Bovine mastitis is a common and economically significant illness that affects dairy cows worldwide (da Costa et al. 2021). This condition is defined by the infection of the mammary glands, which leads to various alterations in the glandular tissues and milk components. These alterations might be physical, chemical, pathological and bacteriological in nature. The detrimental effects of this phenomenon on animal health and welfare, as well as milk quality and quantity, result in significant economic losses (Leghari et al. 2023). Mastitis is a substantial and expensive affliction in the global dairy business. The financial impact of mastitis is significant, with an estimated yearly cost of €124 (roughly $147) per cow. This results in substantial financial losses: €500 million in Germany, €3.125 billion in the European Union and an astonishing €125 billion worldwide. The worldwide incidence of bovine mastitis normally ranges from 30% to 50% of all cows annually (Kabelitz et al. 2021). Bovine mastitis is largely classified according to clinical (or subclinical) features and causes (non‐infectious and infectious). The most prevalent causes of illness are infectious agents, with bacterial infections being the most frequent occurrence among groups of animals (Vidal Amaral et al. 2022). Bacterial pathogens are categorized into contagious, environmental and opportunistic groups. Various research has shown that the most often found bacteria in instances of mastitis include *Staphylococcus aureus*, *Streptococcus agalactiae*, *Streptococcus uberis*, *Escherichia coli* and *Klebsiella pneumoniae* (Morales‐Ubaldo et al. 2023).

*S. agalactiae* is the primary bacterium responsible for causing intramammary infection, which is a common form of mastitis. It is a Gram‐positive bacterium (Yang et al. 2013). It is capable of surviving with or without oxygen and has a protective outer layer called a capsule. This bacterium is often referred to as Group B *Streptococcus* (GBS) based on the Lancefield categorization system (Wataradee et al. 2024). The pathogenicity of a bacterium is determined by many virulence factors. In the case of *S. agalactiae*, these factors include effective adsorption, resistance to phagocytosis and methods to evade the immune system (Liu and Liu 2022). *S. agalactiae* may enhance its capacity to penetrate and colonize its host via the presence of several surface proteins and endotoxins, such as hemolysins and the Christie–Atkins–Munch–Peterson (CAMP) factor. In addition, certain pathogenic elements such as fibrinogen binding (fbs A/B) proteins, adhesion (lmb) proteins and enolase proteins have the ability to harm host tissues and disrupt the immune system, leading to destruction (Hernandez et al. 2021). The C5a peptidase, which is a serine protease, is encoded by the *scpB* gene. It plays a role in binding fibronectin and specifically deactivates the C5a component of the human complement system. The external proteins of *S. agalactiae* consist of the Sip, C and Rib proteins (Baron et al. 2007). The C protein is composed of two antigens, αC and βC. The αC antigen, which is produced by the *bca* gene, plays a role in the entry of pathogens into the cells that make up the surface of the host's body. The βC antigen, which is produced by the *bac* gene, has the ability to attach to IgA antibodies and human factor H (Areschoug et al. 2002). The presence of capsular polysaccharides in *S. agalactiae* is a significant component contributing to its pathogenicity. This polysaccharide exhibits distinct antigenic properties and diverse characteristics that are valuable for serotyping. Previous research has shown that *S. agalactiae* may be classified into 10 distinct kinds (Ia, Ib, II–IX) based on the composition of its capsular polysaccharide (Foxman et al. 2007; Bianchi‐Jassir et al. 2020). These virulence factors enhance the ability of germs to survive and propagate, causing significant harm to the health of animals and people. Performing serotyping on *S. agalactiae* isolates is crucial for comprehending the local epidemiology and for monitoring any instances of serotype replacement or capsular shift. The serotype distribution exhibits variability across different countries, suggesting that a universal approach may not be suitable for comprehending and preventing *S. agalactiae* infections. Hence, it is essential to comprehend the serotyping of *S. agalactiae* and the genetic characterization of virulent strains in order to develop enhanced methods for controlling mastitis (Vaz et al. 2023).

The primary approach for managing the bacterium in dairy cows and human illnesses is by the use of antimicrobial treatment (Singh et al. 2023). Antibiotic resistance is a significant issue in both human and veterinary medicine. At now, there is little official data about the use of various antibiotics in veterinary care and, therefore, the resistance patterns of animal diseases that are prevalent worldwide (Yapicier et al. 2021). Strain characterization and monitoring are crucial for gathering data that enables the assessment of the extent and progression of antibiotic resistance. Therefore, it is crucial to conduct investigations on the antimicrobial activity of mastitis pathogens in order to effectively reduce generated resistance and gather valuable information for making treatment choices. The increase of drug‐resistant strains has led to the intensification of the use of antibiotics, which causes the double concern of environmental pollution and endangering human well‐being. Therefore, the aim of this study was to collect basic information for the prevention and management of bovine mastitis. This was achieved by investigating the prevalence of *S. agalactiae* infection in dairy cows in Chaharmahal and Bakhtiari province, evaluating the resistance of isolated strains to drugs and determining the distribution of virulence genes in these strains. Furthermore, the identification of capsular strains of isolates obtained from infected cows was conducted.

## 2 Materials and Methods

### 2.1 Ethical Issues

Milk samples were collected from cows with clinical mastitis. Prior to sample collection, informed consent was obtained from the herd managers. The study protocol was approved by the Ethics Committee of the Faculty of Veterinary Medicine, Shahrekord Islamic Azad University. The procedure was authorized by the proprietors of the dairy farms under scrutiny. Every possible effort was made to lessen the anguish experienced by animals.

### 2.2 Sampling and Isolation of *S. agalactiae*

Milk samples from 425 mastitic cows were obtained aseptically in 30 dairy farms of Chaharmahal and Bakhtiari province in Iran between March 2023 and March 2024. All cows were milked three times daily and were housed in dry‐lot pens or free stalls. The average monthly bulk tank SCC of cooperating dairies, reported by the processor, was less than 300,000 cells/mL during the sampling period. Clinical mastitis was identified by the California mastitis test (CMT) and clinical examination. Approximately 5 mL of milk were collected in sterile glass bottles, stored in a cool box and transported for culturing. Milk samples were spread onto Trypticase Soy Agar (TSA) plates enriched with 5% bovine blood and incubated at 37°C in a 5% CO_2_ atmosphere. After 24–48 h, the plates were examined for colony characteristics, including morphology, pigmentation and hemolytic activity. Presumptive colonies of *Streptococcus* species were selected and streaked into a blood agar for 24 h for biochemical tests and Gram staining. Catalase, NaCl, bile‐esculin, CAMP, hippurate hydrolysis and sorbitol tests were carried out as described by the National Mastitis Council (2017). Sixty‐eight isolates were identified as *S. agalactiae*, being Gram‐positive cocci, CAMP reaction‐positive and catalase and esculin activity‐negative and stored at −20°C. The *S. agalactiae* strains that were discovered were then preserved in a cation‐adjusted Mueller Hinton broth containing 20% glycerol at a temperature of −80°C.

### 2.3 Pattern of Sensitivity to Antimicrobial Agents

The agar Kirby–Bauer disk diffusion susceptibility technique was used to perform the antimicrobial susceptibility test, according to the recommendations outlined in the VET 01 supplement from the Clinical and Laboratory Standards Institute (CLSI 2018). The CLSI VET01 provides guidelines for evaluating the efficacy of antimicrobial disks and dilution susceptibility tests for microorganisms obtained from animals (Genovese et al. 2020). A total of 16 antimicrobials, belonging to 7 different groups, were chosen specifically for their efficacy in treating bovine mastitis. The antibacterial disks used in the present investigation were ampicillin (AM, 10 µg), azithromycin (AZM, 15 µg), cefalotin (CF, 30 µg), cefepime (FEP, 30 µg), cefotaxime (CTX, 30 µg), ceftriaxone (CRO, 30 µg), chloramphenicol (C, 30 µg), clarithromycin (CLR, 15 µg), erythromycin (E, 15 µg), clindamycin (CC, 2 µg), levofloxacin (LEV, 5 µg), meropenem (MEN, 10 µg), ofloxacin (OFX, 5 µg), penicillin (P, 10 µg), tetracycline (TE, 30 µg) and vancomycin (V, 30 µg). These disks were commercially obtained from Pars Peyvand, Iran. To summarize, GBS colonies were combined with sterile physiological saline and modified to reach a turbidity level corresponding to a concentration of roughly 1.5 × 10^8^ CFU/mL. Next, a sterile cotton swab was immersed in the bacterial solution and applied onto the surface of Mueller–Hinton agar (MHA) plates that contained 5% defibrinated sheep blood. The plates were inoculated with antibiotic disks and then subjected to incubation for a duration of 24 h at a temperature of 37°C in an environment containing 5% CO_2_. After the period of incubation, the area where the growth of bacteria was prevented surrounding the disks was measured using a ruler that has been calibrated and the results were analysed using a standard chart.

### 2.4 Molecular Characterization

#### 2.4.1 DNA Extraction

The DNA template was obtained by boiling bacterial colonies suspended in sterile water for 10 min. The pure DNA concentration was measured through a NanoDrop (MaestroNano Spectrophotometer, MN‐917, Taiwan) and subsequently kept at −20°C for future research.

#### 2.4.2 Determination of Virulence Genes, Antibiotic Resistance Patterns and Serotyping of Selected Isolates Using PCR

To confirm the species identification, a region of the monocopy regulatory gene *dltR*, specific to *S. agalactiae*, was amplified (Lamy et al. 2006). The isolates were also classified using serogrouping, which included determining the particular capsular polysaccharides (Ia, Ib, II, III, IV, V, VI, VII and VIII). All of the isolates were examined for the presence of virulence genes (*cfb*, *fnbB*, *pavA*, *lmb*, *scpB*, *bca*, *atr* and *dlts*) and antibiotic resistance genes (*tetM, tetO, tetT, tetL, tetK, tetS, ermA, ermB, mefA* and *linB*) via gene‐specific primers as outlined in Table 1. Concisely, a PCR mixture of 10 µL was prepared. It consisted of 5 µL of PCR Taq DNA Polymerase Master Mix RED 2x (Ampliqon, Denmark), 1 µL of the DNA template and 0.5 µmol L^−1^ of both the forward primer and the reverse primer. The amplification was carried out utilising a thermal cycler (FlexCycler^2^, Germany). The PCR cycling parameters consisted of an initial denaturation step at 96°C for 1 min, followed by denaturation at 96°C for 10 s, annealing at a temperature between 50°C and 63°C (depending on the melting temperature of each primer) for 30 s, and elongation at 72°C for 30 s (based on the amplicon size of the target gene). These steps were repeated for 30 cycles. The PCR results were seen using a 2% agarose gel that was dyed with DNA‐safe Stain (Cinaclone, Iran).

**TABLE 1:** PCR primers were used to detect and classify *S. agalactiae* isolates obtained from the bovine clinical mastitis.

Target	Gene name	Sequence (5′–3′)	Annealing temperature (°C)	Product size	Reference
Virulence factors	*cfb*	F: TTTCACCAGCTGTATTAGAAGTA R: GTTCCCTGAACATTATCTTTGAT	60	153	Zastempowska et al. (2022)
	*fnbB*	F: TGATGCTGCAAAAGAATTGC R: TTACAGCCCCTTTTTGAGGA	53	629	Momtaz et al. (2017)
	*pavA*	F: TTCCCATGATTTCAACAACAAG R: AACCTTTTGACCATGAATTGGTA	58	498	Shome et al. (2012)
	*lmb*	F: AGTCAGCAAACCCCAAACAG R: GCTTCCTCACCAGCTAAAACG	57	397	Shome et al. (2012)
	*scpB*	F: AGTTGCTTCTTACAGCCCAGA R: GGCGCAGACATACTAGTTCCA	58	567	Lin et al. (2021)
	*cba*	F: AAGCAACTAGAAGAGGAAGC R: TTCTGCTCTGGTGTTTTAGG	58	497	Zastempowska et al. (2022)
	*atr*	F: CAA CGA TTC TCT CAG CTT TGT TAA R: TAA GAA ATC TCT TGT GCG GAT TTC	55	534	Shrestha et al. (2020)
	*dlts*	F: AGGAATACCAGGCGATGAACCGAT R: TGCTCTAATTCTCCCCTTATGGC	56	952	Mousavi et al. (2016)
Antibiotic resistance	*tetM*	F: AGTTTTAGCTCATGTTGATG R: TCCGACTATTTGGACGACGG	51	1826	Zakerifar et al. (2023)
	*tetO*	F: AACTTAGGCATTCTGGCTCAC R: TCCCACTGTTCCATATCGTCA	55	515	Zakerifar et al. (2023)
	*tetT*	F: GGCTCTCATACTGAATGCCAC R: CAGTGGGAATATAAGGACACGTC	53	644	Poyart et al. (2003)
	*tetL*	F: TGAACGTCTCATTACCTG R: ACGAAAGCCCACCTAAAA	50	993	Ding et al. (2016)
	*tetK*	F: TCCTGGAACCATGAGTGT R: AGATAATCCGCCCATAAC	50	198	Ding et al. (2016)
	*tetS*	F: CTCTATGGACAACCCGACAGAAG R: CGCTACATTTGCGAGACTCAG	52	569	Poyart et al. (2003)
	*ermA*	F: ATGAGTCAACGGGTGAATGCT R: GGTGAAAATATGCTCGTGGCA	52	343	Kamińska et al. (2024)
	*ermB*	F: TGGTTTTTGAAAGCCATGCGTCTGA R: GGAACATCTGTGGTATGGCGGGTAAGTT	60	211	Kamińska et al. (2024)
	*mefA*	F: AGTATCATTAATCACTAGTGC R: TTCTTCTGGTACTAAAAGTGG	50	346	Zakerifar et al. (2023)
	*linB*	F: CCTACCTATTGTTTGTGGAA R: ATAACGTTACTCTCCTATTC	54	456	Saed and Ibrahim (2020)
Capsular serotypes	*Ia*	F: GGTCAGACTGGATTAATGGTATGC R: GTAGAAATAGCCTATATACGTTGAATGC	55	521 and 1826	Momtaz et al. (2017)
	*Ib*	F: TAAACGAGAATGGAATATCACAAACC R: GAATTAACTTCAATCCCTAAACAATATCG	53	770	Momtaz et al. (2017)
	*II*	F: GCTTCAGTAAGTATTGTAAGACGATAG R: TTCTCTAGGAAATCAAATAATTCTATAGGG	60	397	Mousavi et al. (2016)
	*III*	F: TCCGTACTACAACAGACTCATCC R: AGTAACCGTCCATACATTCTATAAGC	63	1826	Mousavi et al. (2016)
	*IV*	F: GGTGGTAATCCTAAGAGTGAACTGT R: CCTCCCCAATTTCGTCCATAATGGT	58	578	Mousavi et al. (2016)
	*V*	F: GAGGCCAATCAGTTGCACGTAA R: AACCTTCTCCTTCACACTAATCCT	61	701	Mousavi et al. (2016)
	*VI*	F: GGACTTGAGATGGCAGAAGGTGAA R: CTGTCGGACTATCCTGATGAATCTC	57	487	Mousavi et al. (2016)
	*VII*	F: CCTGGAGAGAACAATGTCCAGAT R: GCTGGTCGTGATTTCTACACA	52	371	Mousavi et al. (2016)
	*VIII*	F: AGGTCAACCACTATATAGCGA R: TCTTCAAATTCCGCTGACTT	55	282	Mousavi et al. (2016)

### 2.5 Statistical Analysis

Data were transferred to a Microsoft Excel spreadsheet (version 15; Microsoft Corp., Redmond, WA, USA) for analysis. Using statistical software (version 16; SPSS Inc., Chicago, USA), a Chi‐square test analysis was performed and differences were considered significant at values of *p* < 0.05.

## 3 Results

### 3.1 Prevalence of *S. agalactiae* in Collected Milk Samples

During a period of one year, a total of 425 samples were collected from 30 dairy farms in the Chaharmahal and Bakhtiari province. Among these samples, 29 (6.82%) strains of *S. agalactiae* were found using biochemical analysis (Table 2). Subsequently, using species‐specific PCR (*dltR* gene amplification), it was proven that all 29 isolates obtained from the initial samples belonged to the *S. agalactiae* species. Serotype III was the most prevalent among the isolates, accounting for 41.37% (12 out of 29 samples). The serotypes Ia, IV and V were the second most prevalent, with each serotype accounting for 17.24% (5 out of 29 isolates). None of the serotypes Ib, II, VI, VII and VIII were detected. In addition, among the 29 known isolates, 2 (6.89%) strains were identified without any serotype. From a statistical seeing, significant differences were found between the frequency of capsular serotypes III and other serotypes (*p* < 0.05).

**TABLE 2:** Distribution of capsular polysaccharide serotypes in *S. agalactiae* strains isolated from bovine mastitis.

Capsular typing	Number of positive isolates	Percentage (%)
Ia	5	17.24
Ib	0	0
II	0	0
III	12	41.37
IV	5	17.24
V	5	17.24
VI	0	0
VII	0	0
VIII	0	0
No Type able	2	6.89

### 3.2 Antibiotic Resistance Pattern

In order to determine the antibiotic sensitivity pattern of *S. agalactiae* strains isolated from raw milk, eight different antibiotic classes including β‐lactam, macrolides, cephalosporins, lincosamides, fluoroquinolones, carbapenems, tetracyclines and glycopeptides were used (Table 3). The *S. agalactiae* strains obtained from mastitis milk samples demonstrated complete resistance to penicillin (100%) and tetracycline (100%), and significant resistance to ampicillin (82.75 %) and erythromycin (82.75 %). The β‐lactam category of antibiotics had the greatest prevalence of antibiotic resistance in *S. agalactiae* isolates, namely in raw milk. In addition, the results showed that almost all the isolated strains are sensitive to Clarithromycin (93.11 %), Meropenem (82.76 %) and Ofloxacin (86.21%). Cefepime (58.62 %) and Chloramphenicol (51.72 %) had a modest impact on *S. agalactiae*. From a statistical seeing, significant differences were found between antimicrobial resistant rates to penicillin, tetracycline, ampicillin and erythromycin with other antibiotic resistance discs also significant differences were found between antimicrobial resistant rates to cefepime and chloramphenicol with cefalotin and clarithromycin discs (*p* < 0.05).

**TABLE 3:** Antibiotic resistance pattern of *S. agalactiae* strains isolated from bovine mastitis.

				Phenotype resistance rate (%)			
Antibiotic disc	Abbreviation	Concentration (µg/mL)	Class of antibiotic	*R*	No. of bacteria	*s*	No. of bacteria
Ampicillin	AM	10	beta‐lactam	82.75	24/29	17.25	5/29
Azithromycin	AZM	15	macrolide	27.58	8/29	72.42	21/29
Cefalotin	FEP	30	beta‐lactam	6.89	2/29	93.11	27/29
Cefepime	CTX	30	beta‐lactam	58.62	17/29	41.38	12/29
Cefotaxime	CF	30	beta‐lactam	24.13	7/29	75.87	22/29
Ceftriaxone	CRO	30	beta‐lactam	37.93	11/29	62.07	18/29
Chloramphenicol	C	30	beta‐lactam	51.72	15/29	48.28	14/29
Clarithromycin	CLR	15	macrolide	6.89	2/29	93.11	27/29
Clindamycin	CC	2	lincosamide	24.13	7/29	75.87	22/29
Erythromycin	E	15	macrolide	82.75	24/29	17.25	5/29
Levofloxacin	LEV	5	quinolone	24.13	7/29	75.87	22/29
Meropenem	MEN	10	beta‐lactam	17.24	5/29	82.76	24/29
Ofloxacin	OFX	5	fluoroquinolone	13.79	4/29	86.21	25/29
Penicillin	P	10	beta‐lactam	100	29/29	0	0/29
Tetracycline	TE	30	tetracycline	100	29/29	0	0/29
Vancomycin	V	30	glycopeptide	31.03	9/29	68.97	20/29

*Note*: Total = 29.

Abbreviations: *R*: resistance; *S*: sensitive.

### 3.3 Distribution of Virulence Factors and Antibiotic Resistance Genes in *S. agalactiae* Isolates

**FIGURE 1:**
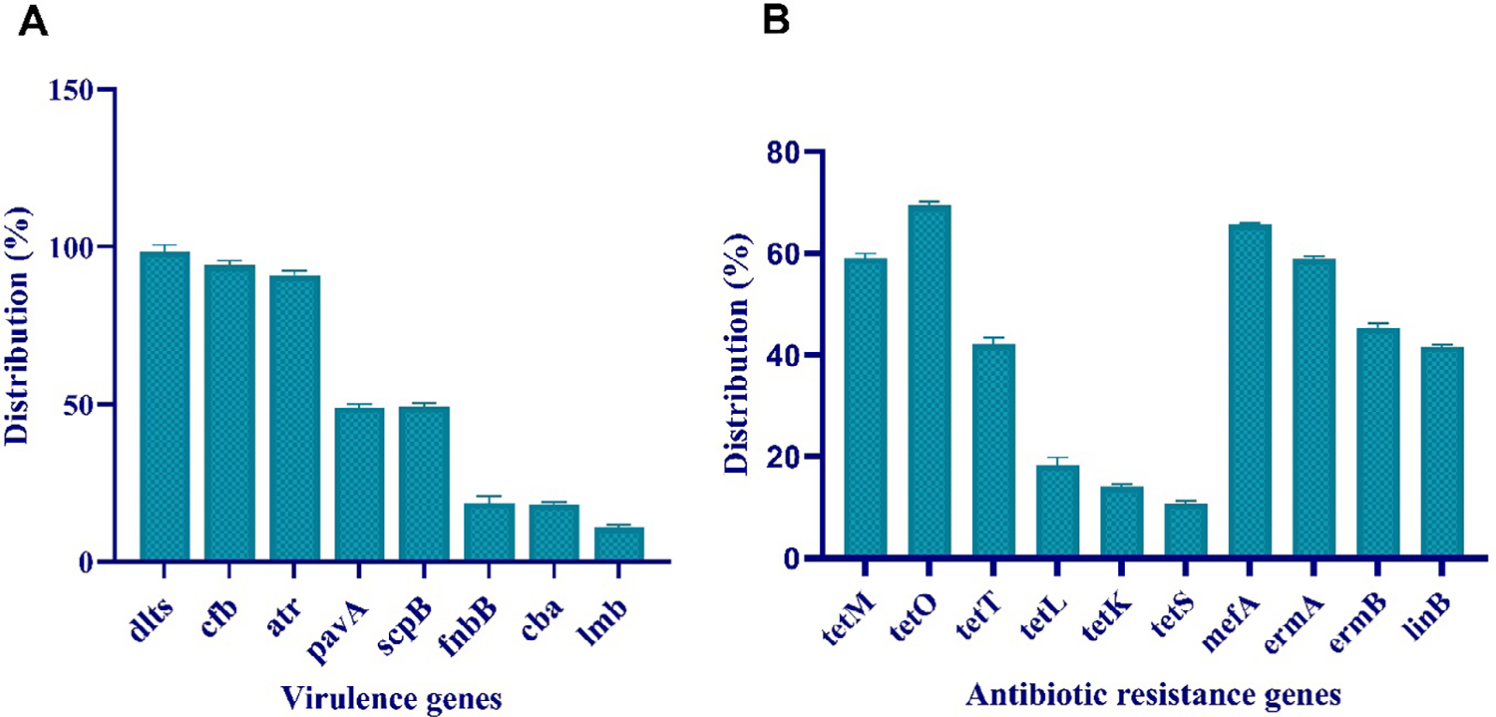
Distribution of virulence genes (A) and antibiotic resistance genes (B) of *S. agalactiae* strains isolated from bovine mastitis.

A total of 29 isolates of *S. agalactiae*, which tested positive for the *dltR* gene, were subjected to conventional PCR using specific primers. Evidence indicates that *dlts* (100%), *cfb* (93.1%) and *atr* (89.65%) genes were extensively prevalent across several *S. agalactiae* isolates (Figure 1A). Among the identified genes, *pavA* was found with 48.27% more among isolates with serotype III. Finally, 48.24% of the identified isolates had the *scpB* gene, while only 10.34% carried the *lmb* gene. From a statistical seeing, significant differences were found between the frequency of *lmb, cba* and *fnbB* genes and other virulence factors (*p* < 0.05).

The frequency of antibiotic resistance genes in the isolated strains was determined using PCR. *tetO* gene was detected in 20 of 29 (68.96%) strains resistant to tetracycline. *tetM* and *tetT* genes were found in 17 (58.62%) and 12 (41.37%) isolates, respectively*. ermA* and *ermB* genes were found in 52 (58.62%) and 45 (44.82 %) *S. agalactiae* strains, respectively. *tetO* (68.96%), *mefA* (65.51%) and *ermA* (58.62%) genes showed the highest frequency among the *S. agalactiae* strains. After that, *tetT* (41.37%), *ermB* (44.82 %) and *linB* (41.37%) genes had moderate frequency among *S. agalactiae* isolates (Figure 1B). From a statistical seeing, significant differences were found between the frequency of *tetS*, *tetK* and *tetL* genes and other antibiotic resistance genes (*p* < 0.05). *tetS* (10.34%), *tetK* (13.79%) and *tetL* (17.24) genes were respectively the least frequent among *S. agalactiae* isolates.

## 4 Discussion

Bovine mastitis is a multifaceted condition with a diverse range of causes, including bacterial, viral and fungal pathogens. Nevertheless, several investigations consistently demonstrate that bacteria remain the primary cause of bovine mastitis. Bovine mastitis frequently occurs by S*. agalactiae*, a highly infectious obligate bacterial infection that infects the mammary gland, leading to milk contamination and posing a possible risk to human health (Han et al. 2022). Research on *S. agalactiae* as the causative agent of bovine mastitis has been conducted worldwide (Jin et al. 2022). Prior investigations have revealed that Denmark and Norway have experienced a higher incidence of bovine mastitis outbreaks caused by *S. agalactiae* compared to other regions (Churakov et al. 2021). In addition, *S. agalactiae* is the predominant pathogen responsible for bovine mastitis in China (Tian et al. 2019). However, there is no accurate information about the prevalence of this bacterium in dairy farms in Iran, especially in Chaharmahal and Bakhtiari province. A total of 425 milk samples were obtained from dairy cows diagnosed with clinical mastitis in various regions of Chaharmahal and Bakhtiari province for the purpose of this research. A total of 29 strains of *S. agalactiae* were obtained from these samples, resulting in an isolation rate of 6.82%. Using normal microbiological procedures, biochemical testing and standard PCR, a total of 29 isolates were identified as *S. agalactiae* in this investigation. The detection approach used in this investigation was suboptimal, however it is often utilized in several commercial detection labs.

This research aimed to investigate all *S. agalactiae* isolates obtained from cows with clinical mastitis for the presence of genes encoding several established and potential virulence factors that are believed to be involved in the pathogenicity of these bacteria. PCR analysis showed the presence of virulence genes *cfb*, *atr*, *scpB* and *pavA* in most isolates, but *cba*, *fnbB* and *lmb* genes were present at a lower rate. In addition, the *dlts* gene was detected in 100% of *S. agalactia* isolates. The high frequency of these genes in *S. agalactiae* was previously described (Shi et al. 2023). GBS may be first recognized by a positive result of a CAMP test, which detects the diffusing CAMP component generated by the majority (95% to 97%) of *S. agalactiae* isolates (Thomas and Cook 2020). The CAMP factor‐encoding gene (*cfb*) is often targeted in PCR experiments and is believed to be found in all GBS strains (Guo et al. 2019). However, new research has identified an *S. agalactiae* isolate that lacks the *cfb* gene (Zhang et al. 2019). Our independent investigation also revealed that the *cfb* gene was present in 93.1% of the *S. agalactiae* strains. Similar findings have been replicated in other investigations concerning *S. agalactiae* in bovine clinical mastitis (Zastempowska et al. 2022). However, several investigators have documented the existence of the *cfb* gene in 98.8% of isolates derived from milk samples of clinical mastitis (Ntozini 2023). *dltS* is a gene that encodes a putative histidine kinase consisting of 395 amino acids. The gene is a component of the dlt operon, which is accountable for the process of D‐alanylation of cell wall teichoic acid (TA). This mechanism exhibits an antagonistic effect towards cationic antimicrobial peptides (AMPs), hence conferring resistance to AMPs on the cell (Du et al. 2023). The results showed that 100% of the strains isolated from the raw milk of cows affected by mastitis contain the *dlts* gene, which can lead to the development of resistance to various classes of antibiotics. The *atr* gene is a crucial element for the virulence of *S. agalactiae* since it facilitates the entry, replication and long‐term survival of the bacterium inside a host (Mousavi et al. 2016). The research discovered that primers designed to amplify the *atr* gene are more effective for diagnosing *S. agalactiae* using PCR techniques compared to primers based on *16S rRNA* genes (Mudzana et al. 2021). In this investigation, the *atr* gene amplification demonstrated a sensitivity of 89.65% (26/29), which aligns with the findings of previous studies conducted by Gavino and Wang (2007), De‐Paris et al. (2011) and Alfa et al. (2010). These studies showed sensitivities of 95.8%, 100% and 90.5% respectively. The additional virulence genes detected in 48.28% of the isolates evaluated in our investigation involve the *scpB* gene, which produces C5a peptidase, a serine protease that deactivates the C5a fraction of human complement, and the *pavA* gene, which encodes a fibronectin‐binding protein that is necessary for pathogenicity (El‐Lakany et al. 2023; Xie et al. 2023). The *cba* gene was detected at comparable rates to earlier investigations conducted in the USA and Sweden, with 20% and 12% of GBS strains carrying the *cba* gene, respectively (Mudzana et al. 2021).

The prevalence of *S. agalactiae* in clinical mastitis samples fluctuates based on geographic location and considering the specific climatic and breeding conditions of each region. Variations in prevalence rates may be seen in different regions within a country. In our analysis, 41.37% of the strains showed capsular Type III. Serotype II of *S. agalactiae* was the most common strain found in dairy cattle in Jiangsu, China, according to a study by Wang Dan et al. (2018). However, a study by Rogers et al. found that capsular serotypes isolated from infants and pregnant women were predominantly Type III (Rogers et al. 2018). In our study, we observed that capsular serotypes in dairy cows were mainly Type III. Based on our findings, we conclude that although serotype II is more common in Jiangsu, China, serotype III is the most common type in dairy cattle in Iran. Table 2 demonstrates that serotype III is the most widespread, accounting for 41.37 % of the cases. Serotypes Ia, IV and V follow with a prevalence of 17.24%, while serotypes Ib, II, VI, VII and VIII are not present.

Antibiotics continue to be the favoured method of therapy for bovine mastitis. Nevertheless, in recent years, the increasing misuse of antibiotics has led to a growing severity of issues related to drug residues and resistance. These issues not only pose a danger to human health but also provide new obstacles to the management of bovine mastitis (Hayes et al. 2020). The research shows that *S. agalactiae* exhibits significant resistance to antibiotics, including ampicillin (82.75%), erythromycin (82.75%) and cefepime (58.62%) while displaying a high susceptibility to meropenem (82.76%), ofloxacin (86.21%) and vancomycin (68.97%). In addition, our findings showed that all bacteria isolated from bovine mastitis milk samples have 100% resistance to penicillin and tetracycline, which highlights the importance of this issue of antibiotic resistance in Chaharmahal and Bakhtiari province. Penicillin used to be the preferred choice for the prevention and treatment of Group B streptococcal infections. However, since 1994, GBS resistance to penicillin has increased (Seki et al. 2015). Tetracyclines are a broad‐spectrum category of antibiotics extensively utilized in both veterinary and human medicine. Although they have proven successful in treating several bacterial infections, their prophylactic use raises considerable concerns. Historically, tetracyclines were utilized as growth promoters in livestock; however, this practice has been prohibited in numerous countries due to apprehensions regarding antibiotic resistance. They are occasionally employed to avert infections in livestock, such as respiratory ailments, yet this may facilitate the emergence of antibiotic‐resistant bacteria (Kosenko et al. 2021). The excessive use of antibiotics, even for prophylaxis, may result in the development of drug‐resistant bacteria. These resistant strains are arduous to treat, complicating infection management. The use of antibiotics in animals may provide unforeseen repercussions for human health (Morley et al. 2005). Antibiotic‐resistant bacteria may be transmitted from animals to humans via the food chain or via direct contact. Contemporary best practices underscore the need to administer antibiotics alone when indispensable and under the supervision of experienced specialists (veterinarians or doctors) (Di Cerbo et al. 2019).

Similar studies showed that *Streptococci* showed significant resistance to enrofloxacin, erythromycin, lincomycin and penicillin (Tian et al. 2019). The results align with a study conducted in Ukraine by Elias et al., where they identified *S. agalactiae* strains that exhibited significant resistance to β‐lactam antibiotics (Elias et al. 2020). Similarly, a study in North China by Tian et al. found streptococci strains with a 100% resistance rate to penicillin and a median sensitivity rate of 92.86% to aminoglycosides (Tian et al. 2019).

Prior research has shown that *S. agalactiae* has significant resistance to β‐lactams, macrolides and lincosamides while displaying considerable susceptibility to quinolones and tetracyclines (López et al. 2018). This work included the analysis of the resistance genes of GBS to several antibiotics. Specifically, we examined the genes responsible for resistance to erythromycin/clindamycin (*ermAM*, *ermB*, *mefA*), tetracycline (*tetM, tetO, tetL, tetT, tetK, tetS*) and lincosamides (*LinB*). In contrast to the findings reported in previous studies, our own data demonstrated that all *S. agalactiae* strains exhibited complete resistance to tetracycline. This resistance may be attributable to the significant prevalence of *tetM* (58.62%), *tetO* (68.96%) and *tetT* (41.37%) genes. The high resistance to tetracycline is partly attributed to the antibiotic's affordability and widespread usage as a preventive measure in the treatment of both animal and human illnesses. Most of our isolates had *ermA* (58.62%), *ermB* (44.82%) and *mefA* (65.51) genes. GBS resistance to macrolides is growing globally and previous studies have shown the involvement of *ermB*, *ermA* and *mefA/E* genes in macrolide resistance (Motallebirad et al. 2021). Macrolide resistance is caused by two main mechanisms: the presence of a ribosome methylase, which is represented by the *erm* genes, and the activity of an efflux pump facilitated by a membrane‐bound protein produced by the *mef* gene (Qian et al. 2023). The resistance to clindamycin is caused by a *linB* gene that encodes a 3‐lincosamide O‐nucleotidyltransferase gene (Yang et al. 2024). The investigation revealed that the primary gene responsible for drug resistance in *S. agalactiae* was *ermB*. Furthermore, there was a significant correlation between the resistance phenotype and the carrier rate for macrolides, lincosamides and tetracyclines. Bacterial strains are known to be resistant to routinely used antibiotics. The low occurrence rate of *tetL* (17.24%) and *tetS* (10.34%) demonstrates that this gene is uncommon in tetracycline‐resistant GBS isolates. The data on resistance genes and phenotypes suggest that *S. agalactiae* exhibits strong resistance to β‐lactams, tetracycline and macrolides while being sensitive to quinolone and glycopeptide. Therefore, it is suggested that depending on the specific bacteria that causes the infection, other antibiotics should be used as the first‐line treatment. Farm animal disease control often shares active substances with human medicines and excessive use of antibiotics is associated with the risk of the creation of MDR foodborne pathogens (van Duin and Paterson 2016). Another point to keep in mind when considering antibiotic resistance of mastitis pathogens is that the interpretive criteria used for categorizing isolates are based on human data for the majority of compounds tested. They may not accurately reflect the efficacy of the drugs in the treatment of bovine mastitis (Rajala‐Schultz et al. 2004) and therefore, veterinary‐specific breakpoints are necessary (Thomas et al. 2015).

## 5 Conclusion

The findings of our study confirm the widespread presence of several virulence genes and antibiotic resistance genes in *S. agalactiae* strains obtained from dairy cows in Chaharmahal and Bakhtiari province, Iran. These genes were previously identified in isolates from other human and animal diseases. Our research concluded that there is a significant occurrence of multidrug‐resistant *S. agalactiae* in cows affected by mastitis. These streptococci exhibited significant resistance to ampicillin, penicillin, tetracycline and erythromycin. The prevailing genes conferring resistance and pathogenicity were *ermB*, *tetO*, *mefA*, *dlts* and *cfb*, respectively. These data present us with the future challenge of closely monitoring the spread of MDR strains, exploring the molecular mechanisms responsible for antimicrobial resistance, and providing a foundation for the development of diagnostic, prophylactic and therapeutic methods, including the perspective of a vaccine.

## Author Contributions

**Pegah HajiAhmadi**: methodology. **Hassan Momtaz**: supervision, methodology, investigation. **Elahe Tajbakhsh**: writing – original draft, methodology.

## Conflicts of Interest

The authors declare no conflicts of interest.

### Peer Review

The peer review history for this article is available at https://publons.com/publon/10.1002/vms3.70275.

## Data Availability

All data analysed during this study are included in this published article. The research was extracted from the PhD thesis in the field of Microbiology and was ethically approved by the Council of Research of the Faculty of Basic Science, Shahrekord Branch, Islamic Azad University, Shahrekord, Iran (Consent Ref Number IR.IAU.SHK.REC.1402.125). Verification of this research project and the licenses related to the sampling process were approved by Prof. Hassan Momtaz (Approval Ref Number MIC201946).

## References

[vms370275-bib-0001] Alfa, M. J. , S. Sepehri , P. de Gagne , M. Helawa , G. Sandhu , and G. K. Harding . 2010. “Real‐Time PCR Assay Provides Reliable Assessment of Intrapartum Carriage of Group B Streptococcus.” Journal of Clinical Microbiology 48: 3095–3099. 20592137 10.1128/JCM.00594-10 PMC2937730

[vms370275-bib-0002] Areschoug, T. , M. Stålhammar‐Carlemalm , I. Karlsson , and G. Lindahl . 2002. “Streptococcal β Protein Has Separate Binding Sites for Human Factor H and IgA‐Fc∗.” Journal of Biological Chemistry 277: 12642–12648. 11812795 10.1074/jbc.M112072200

[vms370275-bib-0003] Baron, M. J. , D. J. Filman , G. A. Prophete , J. M. Hogle , and L. C. Madoff . 2007. “Identification of a Glycosaminoglycan Binding Region of the Alpha C Protein That Mediates Entry of Group B Streptococci Into Host Cells.” Journal of Biological Chemistry 282: 10526–10536. 17259175 10.1074/jbc.M608279200

[vms370275-bib-0004] Bianchi‐Jassir, F. , P. Paul , K.‐N. To , et al. 2020. “Systematic Review of Group B Streptococcal Capsular Types, Sequence Types and Surface Proteins as Potential Vaccine Candidates.” Vaccine 38: 6682–6694. 32888741 10.1016/j.vaccine.2020.08.052 PMC7526974

[vms370275-bib-0005] Churakov, M. , J. Katholm , S. Rogers , R. R. Kao , and R. N. Zadoks . 2021. “Assessing Potential Routes of Streptococcus Agalactiae Transmission Between Dairy Herds Using National Surveillance, Animal Movement Data and Molecular Typing.” Preventive Veterinary Medicine 197: 105501. 34624567 10.1016/j.prevetmed.2021.105501

[vms370275-bib-0006] CLSI—Clinical and Laboratory Standards Institute . 2018. Performance Standards for Antimicrobial Disk and Dilution Susceptibility Tests for Bacteria Isolated from Animals. 4th ed. Clinical and Laboratory Standards Institute.

[vms370275-bib-0007] da Costa, G. M. , N. A. Ribeiro , M. S. Gonçalves , J. R. da Silva , D. A. da Costa Custódio , and G. F. Mian . 2021. “Antimicrobial Susceptibility Profile of Streptococcus Agalactiae Strains Isolated From Bovine Mastitis.” Brazilian Journal of Veterinary Research and Animal Science 58: e178109.

[vms370275-bib-0008] De‐Paris, F. , A. B. M. P. Machado , T. C. Gheno , B. M. Ascoli , K. R. P. de Oliveira , and A. L. Barth . 2011. “Group B Streptococcus Detection: Comparison of PCR Assay and Culture as a Screening Method for Pregnant Women.” Brazilian Journal of Infectious Diseases 15: 323–327. 21861001

[vms370275-bib-0009] Ding, Y. , J. Zhao , X. He , et al. 2016. “Antimicrobial Resistance and Virulence‐Related Genes of Streptococcus Obtained From Dairy Cows With Mastitis in Inner Mongolia, China.” Pharmaceutical Biology 54: 162–167. 25856704 10.3109/13880209.2015.1025290

[vms370275-bib-0010] Di Cerbo, A. , F. Pezzuto , G. Guidetti , S. Canello , and L. Corsi . 2019. “Tetracyclines: Insights and Updates of Their Use in Human and Animal Pathology and Their Potential Toxicity.” Open Biochemistry Journal 13, no. 1: 1–12.

[vms370275-bib-0011] Du, J. , S. Huang , M. Wu , et al. 2023. “Dlt Operon Regulates Physiological Function and Cariogenic Virulence in Streptococcus Mutans.” Future Microbiology 18: 225–233. 37097048 10.2217/fmb-2022-0165

[vms370275-bib-0012] El‐Lakany, R. R. , E. S. Abdelmaged , M. Shams , R. Hassan , and D. E. Rizk . 2023. “Incidence of Virulence Determinants Among Streptococcus Agalactiae Isolated From Pregnant Women and Association With Their Serotypes.” Egyptian Journal of Basic and Applied Sciences 10: 650–670.

[vms370275-bib-0013] Elias, L. , A. S. Balasubramanyam , O. Y. Ayshpur , et al. 2020. “Antimicrobial Susceptibility of *Staphylococcus aureus*, *Streptococcus agalactiae*, and *Escherichia coli* Isolated From Mastitic Dairy Cattle in Ukraine.” Antibiotics 9: 469. 32752205 10.3390/antibiotics9080469 PMC7459615

[vms370275-bib-0014] Foxman, B. , B. Gillespie , S. Manning , and C. F. Marrs . 2007. “Risk Factors for Group B Streptococcal Colonization: Potential for Different Transmission Systems by Capsular Type.” Annals of Epidemiology 17: 854–862. 17689259 10.1016/j.annepidem.2007.05.014 PMC2099698

[vms370275-bib-0015] Gavino, M. , and E. Wang . 2007. “A Comparison of a New Rapid Real‐Time Polymerase Chain Reaction System to Traditional Culture in Determining Group B Streptococcus Colonization.” American Journal of Obstetrics and Gynecology 197: 388.e1–388.e4. 10.1016/j.ajog.2007.06.016 17904971

[vms370275-bib-0016] Genovese, C. , F. D'Angeli , V. di Salvatore , G. Tempera , and D. Nicolosi . 2020. “ *Streptococcus agalactiae* in Pregnant Women: Serotype and Antimicrobial Susceptibility Patterns Over Five Years in Eastern Sicily (Italy).” European Journal of Clinical Microbiology & Infectious Diseases 39: 2387–2396. 32700131 10.1007/s10096-020-03992-8 PMC7669783

[vms370275-bib-0017] Guo, D. , Y. Xi , S. Wang , and Z. Wang . 2019. “Is a Positive Christie‐Atkinson‐Munch‐Peterson (CAMP) Test Sensitive Enough for the Identification of *Streptococcus agalactiae*?” BMC Infectious Diseases 19: 1–5. 30606123 10.1186/s12879-018-3561-3 PMC6318942

[vms370275-bib-0018] Han, G. , B. Zhang , Z. Luo , et al. 2022. “Molecular Typing and Prevalence of Antibiotic Resistance and Virulence Genes in *Streptococcus agalactiae* Isolated From Chinese Dairy Cows With Clinical Mastitis.” PLoS ONE 17: e0268262. 35522690 10.1371/journal.pone.0268262 PMC9075616

[vms370275-bib-0019] Hayes, K. , F. O'Halloran , and L. Cotter . 2020. “A Review of Antibiotic Resistance in Group B Streptococcus: The Story so Far.” Critical Reviews in Microbiology 46: 253–269. 32363979 10.1080/1040841X.2020.1758626

[vms370275-bib-0020] Hernandez, L. , E. Bottini , J. Cadona , et al. 2021. “Multidrug Resistance and Molecular Characterization of *Streptococcus agalactiae* Isolates From Dairy Cattle With Mastitis.” Frontiers in Cellular and Infection Microbiology 11: 647324. 33996629 10.3389/fcimb.2021.647324 PMC8120232

[vms370275-bib-0021] Jin, Z. , J. Li , H. Zhou , et al. 2022. “Serotype Distribution, Virulence Determinants and Antimicrobial Susceptibility of *Streptococcus agalactiae* Isolated From Young Infants.” Pathogens 11: 1355. 36422606 10.3390/pathogens11111355 PMC9697028

[vms370275-bib-0022] Kabelitz, T. , E. Aubry , K. van Vorst , T. Amon , and M. Fulde . 2021. “The Role of *Streptococcus* Spp. In Bovine Mastitis.” Microorganisms 9: 1497. 34361932 10.3390/microorganisms9071497 PMC8305581

[vms370275-bib-0023] Kamińska, D. , M. Ratajczak , D. M. Nowak‐Malczewska , et al. 2024. “Macrolide and Lincosamide Resistance of *Streptococcus agalactiae* in Pregnant Women in Poland.” Scientific Reports 14: 3877. 38366099 10.1038/s41598-024-54521-y PMC10873391

[vms370275-bib-0024] Kosenko, Y. , S. Bilous , N. Ostapiv , and L. Zaruma . 2021. “Use of Tetracyclines and Sulfonamides for the Treatment of Infectious Diseases in Animals.” ScienceRise: Biological Science 2, no. 27: 10–17. 10.15587/2519-8025.2021.235057.

[vms370275-bib-0025] Lamy, M. C. , S. Dramsi , A. Billoët , et al. 2006. “Rapid Detection of the “Highly Virulent” Group B *Streptococcus* ST‐17 Clone.” Microbes and Infection 8, no. 7: 1714–1722. 16822689 10.1016/j.micinf.2006.02.008

[vms370275-bib-0026] Leghari, A. , S. A. Lakho , F. M. Khand , et al. 2023. “Molecular Epidemiology, Characterization of Virulence Factors and Antibiotic Resistance Profile of *Streptococcus agalactiae* Isolated From Dairy Farms in China and Pakistan.” Journal of Integrative Agriculture 22: 1514–1528.

[vms370275-bib-0027] Lin, L. , X. Huang , H. Yang , et al. 2021. “Molecular Epidemiology, Antimicrobial Activity, and Virulence Gene Clustering of *Streptococcus agalactiae* Isolated From Dairy Cattle With Mastitis in China.” Journal of Dairy Science 104: 4893–4903. 33551160 10.3168/jds.2020-19139

[vms370275-bib-0028] Liu, Y. , and J. Liu . 2022. “Group B *Streptococcus*: Virulence Factors and Pathogenic Mechanism.” Microorganisms 10: 2483. 36557736 10.3390/microorganisms10122483 PMC9784991

[vms370275-bib-0029] López, Y. , E. Parra , V. Cepas , et al. 2018. “Serotype, Virulence Profile, Antimicrobial Resistance and Macrolide‐Resistance Determinants in *Streptococcus agalactiae* Isolates in Pregnant Women and Neonates in Catalonia, Spain.” Enfermedades Infecciosas y Microbiologia Clinica (English Ed.) 36: 472–477. 29029763 10.1016/j.eimc.2017.08.006

[vms370275-bib-0030] Momtaz, H. , R. Soleimani , and A. Jazayeri . 2017. “Prevalence of Virulence Factors and Antimicrobial Resistance of *Streptococcus agalactiae* and *Streptococcus uberis* in Ruminant Sub‐Clinical Mastitic Milk in Iran.” International Journal of Medical Laboratory 4: 34–47.

[vms370275-bib-0031] Morales‐Ubaldo, A. L. , N. Rivero‐Perez , B. Valladares‐Carranza , V. Velázquez‐Ordoñez , L. Delgadillo‐Ruiz , and A. Zaragoza‐Bastida . 2023. “Bovine Mastitis, a Worldwide Impact Disease: Prevalence, Antimicrobial Resistance, and Viable Alternative Approaches.” Veterinary and Animal Science 21: 100306. 37547227 10.1016/j.vas.2023.100306 PMC10400929

[vms370275-bib-0032] Motallebirad, T. , H. Fazeli , D. Azadi , D. Shokri , S. Moghim , and B. N. Esfahani . 2021. “Determination of Capsular Serotypes, Antibiotic Susceptibility Pattern, and Molecular Mechanism of Erythromycin Resistance Among Clinical Isolates of Group B *Streptococcus* in Chaharmahal & Bakhtiari, Iran.” Advanced Biomedical Research 10: 27. 34760809 10.4103/abr.abr_269_20 PMC8531737

[vms370275-bib-0033] Mousavi, S. M. , S. M. Hosseini , R. Y. Mashouf , and M. R. Arabestani . 2016. “Identification of Group B *Streptococci* Using 16S rRNA, Cfb, scpB, and Atr Genes in Pregnant Women by PCR.” Acta Medica Iranica 765–770. 28120587

[vms370275-bib-0034] Morley, P. S. , M. D. Apley , T. E. Besser , et al. 2005. “Antimicrobial Drug Use in Veterinary Medicine.” Journal of Veterinary Internal Medicine 19, no. 4: 617–629. 16095186 10.1892/0891-6640(2005)19[617:aduivm]2.0.co;2

[vms370275-bib-0035] Mudzana, R. , R. T. Mavenyengwa , and M. Gudza‐Mugabe . 2021. “Analysis of Virulence Factors and Antibiotic Resistance Genes in Group B *Streptococcus* From Clinical Samples.” BMC Infectious Diseases 21: 1–11. 33509097 10.1186/s12879-021-05820-6 PMC7844887

[vms370275-bib-0036] National Mastitis Council . 2017. Laboratory Handbook on Bovine Mastitis. 3rd ed. National Mastitis Council.

[vms370275-bib-0037] Ntozini, B. 2023. Molecular Characterization of Invasive Streptococcus Agalactiae in South Africa, 2019–2020. Faculty of Health Sciences, University of the Witwatersrand.

[vms370275-bib-0038] Poyart, C. , L. Jardy , G. Quesne , P. Berche , and P. Trieu‐Cuot . 2003. “Genetic Basis of Antibiotic Resistance in *Streptococcus agalactiae* Strains Isolated in a French Hospital.” Antimicrobial Agents and Chemotherapy 47: 794–797. 12543695 10.1128/AAC.47.2.794-797.2003 PMC151750

[vms370275-bib-0039] Qian, Y. , S. Mobashery , and J. F. Fisher . 2023. “Macrolide, Lincosamide, Glycopeptide, and Other Antibacterial Antibiotics.” In Medicinal Chemistry of Chemotherapeutic Agents: A Comprehensive Resource of Anti‐Infective and Anti‐Cancer Drugs, edited by P. C. Acharya and M. Kurosu , 157–213. Academic Press.

[vms370275-bib-0040] Rajala‐Schultz, P. J. , K. L. Smith , J. S. Hogan , and B. C. Love . 2004. “Antimicrobial Susceptibility of Mastitis Pathogens From First Lactation and Older Cows.” Veterinary Microbiology 102, no. 1‐2: 33–42. 15288925 10.1016/j.vetmic.2004.04.010

[vms370275-bib-0041] Rogers, L. M. , J. A. Gaddy , S. D. Manning , and D. M. Aronoff . 2018. “Variation in Macrophage Phagocytosis of *Streptococcus agalactiae* Does Not Reflect Bacterial Capsular Serotype, Multilocus Sequence Type, or Association With Invasive Infection.” Pathogens & Immunity 3: 63. 29930990 10.20411/pai.v3i1.233 PMC6007880

[vms370275-bib-0042] Saed, H. A. E.‐M. R. , and H. M. M. Ibrahim . 2020. “Antimicrobial Profile of Multidrug‐Resistant *Streptococcus* Spp. Isolated From Dairy Cows With Clinical Mastitis.” Journal of Advanced Veterinary and Animal Research 7: 186. 32607349 10.5455/javar.2020.g409 PMC7320817

[vms370275-bib-0043] Seki, T. , K. Kimura , M. E. Reid , et al. 2015. “High Isolation Rate of MDR Group B *Streptococci* With Reduced Penicillin Susceptibility in Japan.” Journal of Antimicrobial Chemotherapy 70: 2725–2728. 26169560 10.1093/jac/dkv203

[vms370275-bib-0044] Shi, H. , M. Zhou , Z. Zhang , et al. 2023. “Molecular Epidemiology, Drug Resistance, and Virulence Gene Analysis of *Streptococcus agalactiae* Isolates From Dairy Goats in Backyard Farms in China.” Frontiers in Cellular and Infection Microbiology 12: 1049167. 36699728 10.3389/fcimb.2022.1049167 PMC9868259

[vms370275-bib-0045] Shome, B. R. , M. Bhuvana , S. D. Mitra , et al. 2012. “Molecular Characterization of *Streptococcus agalactiae* and *Streptococcus uberis* Isolates From Bovine Milk.” Tropical Animal Health and Production 44: 1981–1992. 22588571 10.1007/s11250-012-0167-4

[vms370275-bib-0046] Shrestha, K. , A. K. Sah , N. Singh , P. Parajuli , and R. Adhikari . 2020. “Molecular Characterization of *Streptococcus agalactiae* Isolates From Pregnant Women in Kathmandu City.” Journal of Tropical Medicine 2020: 4046703. 32908547 10.1155/2020/4046703 PMC7474781

[vms370275-bib-0047] Singh, R. , A. Arora , T. Rai , and M. Chandra . 2023. “Isolation, Antibiogram and Molecular Characterization of Group b *Streptococci* Isolates From Bovine Mastitis.” Indian Journal of Animal Research 57: 114–119.

[vms370275-bib-0048] Thomas, L. , and L. Cook . 2020. “Two‐Component Signal Transduction Systems in the Human Pathogen *Streptococcus agalactiae* .” Infection and Immunity 88, no. 7: e00931–19. 10.1128/iai.00931-19. 31988177 PMC7309623

[vms370275-bib-0049] Thomas, V. , A. de Jong , H. Moyaert , et al. 2015. “Antimicrobial Susceptibility Monitoring of Mastitis Pathogens Isolated From Acute Cases of Clinical Mastitis in Dairy Cows Across Europe: VetPath Results.” International Journal of Antimicrobial Agents 46, no. 1: 13–20. 26003836 10.1016/j.ijantimicag.2015.03.013

[vms370275-bib-0050] Tian, X. , N. Zheng , R. Han , et al. 2019. “Antimicrobial Resistance and Virulence Genes of *Streptococcus* Isolated From Dairy Cows With Mastitis in China.” Microbial Pathogenesis 131: 33–39. 30940606 10.1016/j.micpath.2019.03.035

[vms370275-bib-0051] van Duin, D. , and D. L. Paterson . 2016. “Multidrug‐Resistant Bacteria in the Community: Trends and Lessons Learned.” Infectious Disease Clinics 30, no. 2: 377–390. 27208764 10.1016/j.idc.2016.02.004 PMC5314345

[vms370275-bib-0052] Vaz, M. J. , S. Dongas , and A. J. Ratner . 2023. “Capsule Production Promotes Group B *Streptococcus* Intestinal Colonization.” Microbiology Spectrum 11: e02349–23. 37732775 10.1128/spectrum.02349-23 PMC10655599

[vms370275-bib-0053] Vidal Amaral, J. R. , R. T. Jucá Ramos , F. Almeida Araújo , et al. 2022. “Bacteriocin Producing *Streptococcus agalactiae* Strains Isolated From Bovine Mastitis in Brazil.” Microorganisms 10: 588. 35336163 10.3390/microorganisms10030588 PMC8953382

[vms370275-bib-0054] Wang, D. , F. Yang , X. Li , et al. 2018. “Isolation, Identification and Drug Resistance Detection of the Pathogenic Bacteria Causing Dairy Cow Mastitis in Suzhou and Capsular Polysaccharide Typing Test of the Pathogens.” Chinese Journal of Preventive Veterinary Medicine 40, no. 08: 680–686.

[vms370275-bib-0055] Wataradee, S. , T. Boonserm , S. Samngamnim , and K. Ajariyakhajorn . 2024. “Characterization of Virulence Factors and Antimicrobial Susceptibility of *Streptococcus agalactiae* Associated With Bovine Mastitis Cases in Thailand.” Animals 14: 447. 38338090 10.3390/ani14030447 PMC10854646

[vms370275-bib-0056] Xie, X. , Z. Pan , Y. Yu , et al. 2023. “Prevalence, Virulence, and Antibiotics Gene Profiles in *Lactococcus garvieae* Isolated From Cows With Clinical Mastitis in China.” Microorganisms 11: 379. 36838344 10.3390/microorganisms11020379 PMC9965093

[vms370275-bib-0057] Yang, Y. , Y. Liu , Y. Ding , et al. 2013. “Molecular Characterization of *Streptococcus agalactiae* Isolated From Bovine Mastitis in Eastern China.” PLoS ONE 8: e67755. 23874442 10.1371/journal.pone.0067755 PMC3707890

[vms370275-bib-0058] Yang, Y. , S. Xie , F. He , et al. 2024. “Recent Development and Fighting Strategies for Lincosamide Antibiotic Resistance.” Clinical Microbiology Reviews 37: e00161–23. 10.1128/cmr.00161-23 PMC11237733 38634634

[vms370275-bib-0059] Yapicier, Ö. Ş. , E. Sababoglu , D. Ozturk , H. Turutoglu , F. Pehlivanoglu , and M. Kaya . 2021. “Lancefield Classification and Antimicrobial Resistance of Hemolytic *Streptococci* Isolated From Bovine Mastitis.” Veterinaria Italiana 57: 41–47. 34313097 10.12834/VetIt.1855.9879.3

[vms370275-bib-0060] Zakerifar, M. , H. Kaboosi , H. R. Goli , Z. Rahmani , and F. Peyravii Ghadikolaii . 2023. “Antibiotic Resistance Genes and Molecular Typing of *Streptococcus agalactiae* Isolated From Pregnant Women.” BMC Pregnancy and Childbirth 23: 43. 36658541 10.1186/s12884-023-05380-4 PMC9854082

[vms370275-bib-0061] Zastempowska, E. , M. Twarużek , J. Grajewski , and H. Lassa . 2022. “Virulence Factor Genes and Cytotoxicity of *Streptococcus agalactiae* Isolated From Bovine Mastitis in Poland.” Microbiology Spectrum 10: e02224–21. 10.1128/spectrum.02224-21 PMC9241884 35608349

[vms370275-bib-0062] Zhang, Z. , F. Yang , X.‐P. Li , et al. 2019. “Distribution of Serotypes, Antimicrobial Resistance and Virulence Genes Among *Streptococcus agalactiae* Isolated From Bovine in China.” Acta Scientiae Veterinariae 47: 1699.

